# Behavioral factors for predicting severity of enuresis and treatment responses in different compliance groups receiving behavioral therapy

**DOI:** 10.12669/pjms.334.12922

**Published:** 2017

**Authors:** Yanli Ma, Xiaomei Liu, Ying Shen

**Affiliations:** 1 Yanli Ma, Department of Nephrology, Beijing Children’s Hospital, Capital Medical University, Beijing Key Laboratory of Chronic Kidney Disease andBlood Purification of Children. No.56, South Lishi Road, Xicheng District, Beijing, China; 2 Xiaomei Liu, Department of Nephrology, Beijing Children’s Hospital, Capital Medical University, Beijing Key Laboratory of Chronic Kidney Disease andBlood Purification of Children. No.56, South Lishi Road, Xicheng District, Beijing, China; 3 Ying Shen, Department of Nephrology, Beijing Children’s Hospital, Capital Medical University, Beijing Key Laboratory of Chronic Kidney Disease andBlood Purification of Children. No.56, South Lishi Road, Xicheng District, Beijing, China

**Keywords:** Enuresis, Behavioral factor, Behavioral interventions, Compliance

## Abstract

**Objective:**

To investigate behavioral factors for predicting severity of nocturnal enuresis and compare response rates in different compliance groups of behavioral interventions.

**Methods:**

Three hundred eleven children diagnosed with nocturnal enuresis were enrolled. This study was conducted at Beijing Children’s Hospital affiliated to the Capital Medical University from September 2016 to December 2016. Correlation of severity of enuresis and behavioral factors was investigated. All patients were treated with desmopressin based on behavioral interventions. After twomonth treatment, the patients were grouped according to the compliance of behavioral therapy. Then response rates in different compliance groups were compared.

**Results:**

Multivariate analysis revealed stool frequency, drinking water before going to bed, awaking the child to toilet at night, and appetite were independent risk factors affecting the severity of enuresis. The complete response rate of enuresis and partial response ratein fullcompliance group are higher than thoseof partial compliance and non-compliance group(21.9% *vs* 11.3%, 78.1% *vs* 59.8%; 21.9% *vs* 0%, 78.1% *vs* 49.1%; P<0.01). The complete response rate and partial response rate of partial compliance group are higher than those of the non-compliance group (P<0.01).

**Conclusions:**

Stool frequency, drinking water before going to bed, awaking the child to toilet at night, and appetite are independent predictive factors affecting the severity of enuresis. Good compliance of behavioral interventions may have a crucial role for better therapeutic outcomes.

## INTRODUCTION

Enuresis is defined as repeated voiding of urine into the bed or clothes at least twice per week for more than three months in a child who is more thanfive years of age.^1^ Nocturnal enuresis (NE) occurs in 15% to 25% of children aged five years old. The spontaneous remission rate of enuresis every year is about 15%^2^, but if not treated, especially severe enuresis, can persist indefinitely with prevalence rates of 2%-3% in adulthood.^3^ Children with NE are prone to have mental health problems which are not conducive to children’s physical and mental health.^4^ Psychiatric disorders act as the etiology, complication, or adverse consequences of enuresis. In addition, NE also has brought many problems to the children and their families, such aslow self-evaluation, social frustration, and sleep disturbance.^5^ As a result, more attention is required for NE. Treatment methods of enuresis mainly involve behavioral interventions, enuresis alarm, and medications. Behavioral interventions are the basic therapy for enuresis, including offering suggestions for voiding patterns and frequency (proper voiding posture, regular voiding habits, and regular bowel habits), limiting fluid intake, reward systems, treating constipation, wake-up training, and bladdertraining. Some scholars have discussed the risk factors of the severity of enuresis, but mainly focusing on bladder capacity, urinary osmotic pressure, bladder wall thickness, and demographic indicators.^6^ We now pose the questions: are life and behavioral factors (e.g. stool frequency, appetite, time for bed) the independent risk factors affecting the severity of enuresis before treatment? Whether the therapeutic effect of drugs combined with behavioral interventions is associated with the compliance of behavioral interventions? We have tried to find answers to these questions.

## METHODS

Three hundred eleven children and adolescents diagnosed with NE in Beijing Children’s Hospital affiliated to the Capital Medical University from September 2016 to December 2016 were enrolled in this study. Inclusion criteria: (1) aged 5-15 years old, primary nocturnal enuresis (PNE, two or more wet nights per week and not dry for more than 6 months). (2) not receiving any medication for enuresis during the last 6 months. (3) lack of clinical or laboratory signs suggestive of any underlying disease other than enuresis. This study was conducted in accordance with the regulatory standards of Good Clinical Practice and the Declaration of Helsinki and approved by the Institutional Review Board of Beijing Children’s Hospital.

### Grouped comparison of severity of enuresis

The patients were divided into subgroups according to gender, family history of NE, with or without lower urinary tract symptoms, maternal educational level, family economic conditions, awaken to toilet at night or not, appetite, snoring or not at night, dinner time, time for bed, stool frequency, hard stools or not, whether drinking water before going to bed, and ageof stopping using diapers. Subsequently, theseverity of enuresisamong different groups was compared.

### Hard stools

The Bristol Stool Scale Form (BSFS) has become the most widely used instrument for assessing stool characteristics.^7^,^8^ The BSFS classifies stool forms into 7 categories ranging from hard stools (types 1 and 2) to loose stools (types 6 and 7) with an intermediate category considered as normal stools (types 3, 4, and 5). The BSFS describes type 1 as “separate hard lumps, like nuts” and type 2 as “sausage-shaped but lumpy”. To make it easier for children to understand, the Modified-BSFS describes type 1 as “rabbit droppings” and type 2 as “bunch of grapes”.^9^

### Appetite

One appetite-related variable, “desire to eat”, was involved for evaluation.^10^ The patients have one question to answer: do you have the desire to eat? The answers include: not at all, extremely, and somewhere in between. Subsequently, the patients were divided into groups of good, poor, and medium appetite according to the answers.

### Severity of enuresis

Severity of NE was classified as mild-moderate and severe according to the frequency of enuresis. More than 5 wet nights weekly was classified as severe and 5 or fewer as mild-moderate.^11^

### Treatment and assessment of compliance

All patients were recommended to take oral desmopressin (0.2mg) before going to bed (FerringPharmaceutical Company, Sweden, trade name: minirin, 0.1mg/tablet) based on behavioral interventions. Fluid intake should be reduced from 1 h before desmopressin administration and for 8 h subsequently. Behavioral interventions mainly include:

***(1) Education:*** Pass on knowledge of enuresis to the parents: enuresis has a relatively high incidence--reduce their guilt and self-blame. The spontaneous cure rate of enuresis is relatively high.

***(2) Life-style advice:*** Get into a good habit of going to bed and getting up early: go to bed before 9 pm o’clock and empty the bladder before going to bed. Do not have to limit water intake during the daytime to ensure normal daily fluid intake. Limiting fluid intake is necessary within 3 hours before bedtime. Healthy eating habits include: reduce the intakes of spicy, fried, cold foods, caffeine, and snacks, eat more fruits and vegetables. Develop the habit ofregular bladder and bowel emptying.

***(3) Encouragement andreward systems:*** Explain to the children patiently that enuresis is not their faults and the disease is curative. The child might receive a star for every dry night, and a reward after a preset number of stars have been earned.

***(4) Wake-up training:*** According to the time rules of bed-wetting, awake the child to void before enuresis; orwhen the child suddenly turned around in a quiet sleep, waking the child to get up and urinate.

***(5) Bladder training:*** All patients were required to recorddaytime and overnight bladder diariesdaily. Training children to regular voiding habits and right voiding posture;attempting to increase the functional bladder capacity by encouraging children to drink more water and delaying urination for extended periods of time during the day, two times/day;teaching children to interrupt their stream of urine in order to strengthen their pelvic muscle.

Full compliance was defined if all of the above five aspects of behavioralinterventions completed daily, three or four aspects completedwas defined as partial compliance, andtwo or fewer aspectscompleted as non-compliance. After two month treatment, children with PNE were grouped according to the different compliances. Then, the comparison of response rates in three different groups was made.

### Effect evaluation criteria

The number and percentage of non-responders, partialresponders, and fullresponders were evaluated after two-month therapy in each group. Nonresponse is defined as a 0%–49% decrease. Partial response is defined as a 50%–99% decrease. Full response is defined as a 100% reduction.^12^

### Statistical analysis

The collected data was analyzed through descriptive analysis and using the chi-square and t-tests. P value <0.05 was considered statistically significant. A logistic regression model was used to investigate the relationship between the severity of enuresis and possible predictive variables.

## RESULTS

### Of the 311 initially enrolled patients, 11 were excluded for the following reasons

Onepatient did not meet the inclusion criteria, one did not sign informed consent, and nine were lost to follow-up. Thus, 300 children were included in this study. Of these, 158 were male and 142 were female, aged between 5 and 15 years (mean age, 7.25±2.39 years old). Baseline clinical characteristics of the patients were shown in Table-I.

**Table-I:** Baseline clinical characteristics and demographic features.

*Variables*		*N*	*Percentage (%)*
Gender	Male	158	52.6
	Female	142	47.3
Lower urinary tract symptoms	Without	94	31.3
	With	206	68.7
Family history of enuresis	No	207	69.0
	Yes	93	31.0
Maternal educational level	Middle school	131	43.7
	University degree	141	47.0
	Graduate	28	9.3
Family economic conditions	Good	45	15.0
	Medium	214	71.3
	Poor	41	13.7
Age (years, Mean±SD)	7.25±2.39		
Frequency of NE (times/week, Median, interquartile range)	7(5.25-7.00)		

### Grouped comparison of the severity of enuresis

The results showed that appetite, time for bed, snoring, stool frequency, hard stool, drinking water before going to bed, and awaking the child to toilet at night are related with the severity of enuresis(Table-II).

**Table-II:** Comparison of severity of enuresis in different groups.

*Variables*		*Mild to moderate (93)*		*Severe (207)*		*P value*

		*N*	*%*	*N*	*%*	
Gender	Male	46	29.1	112	70.9	0.517
	Female	36	25.4	106	74.6	
Family history of enuresis	No	60	29.0	147	71.0	0.401
	Yes	22	23.7	71	76.3	
Lower urinary tract symptoms	Without	29	30.9	65	69.1	0.402
	With	53	25.7	153	74.3	
Family economic conditions	Good	16	35.6	29	64.4	0.402
	Medium	55	25.7	159	74.3	
	Poor	11	26.8	30	73.2	
Maternal educational level	Middle school	34	26.0	97	74.0	0.894
	University degree	40	28.4	101	71.6	
	Graduate	8	28.6	20	71.4	
Stool frequency	1-2days/time	78	37.1	132	62.9	0.000
	≥3days/time	4	4.4	86	95.6	
Hard stool	No	78	37.0	133	63.0	0.000
	Yes	4	4.5	85	95.5	
Appetite	Good	59	73.8	21	26.2	0.000
	Medium	16	19.8	65	80.2	
	Poor	7	5.0	132	95.0	
Dinner time	Before 6 p.m.	61	31.1	135	68.9	0.056
	After 6 p.m.	21	20.2	83	79.8	
Time for bed	Before 9 p.m.	61	36.3	107	63.7	0.000
	After 9 p.m.	21	15.9	111	84.1	
Drinking water before going to bed	No	52	61.9	32	38.1	0.000
	Yes	30	13.9	186	86.1	
Snoring or not	No	71	33.3	142	66.7	0.004
	Yes	11	12.6	76	87.4	
Awake the child to toilet at night	Yes	34	43.6	44	56.4	0.000
	No	48	21.6	174	78.4	
Age of stopping using diapers	Before 2 years old	54	27.0	146	73.0	0.891
	After 2 years old	28	28.0	72	72.0	

### Multivariate analysis of predictive factors of the severity of enuresis

With 7 significant factors in group comparison (appetite, time for bed, snoring, stool frequency, hard stool, drinking water before going to bed, and awaking the child to toilet at night) as independent variables and the severity of enuresis as dependent variables (1=mild to moderate; 2= severe), a logistic regression model was used to investigate the relationship between the severity of enuresis and possible predictive variables. The results were shown in Table-III.

**Table-III:** Multivariate analysis of the severity of enuresis

*Factors*	*B*	*Standard error*	*Wald* value	*P*	*OR*	*95% confidence interval*	

						*Lower limit*	*Upper limit*
Awake the child to toilet at night	1.446	0.405	12.768	0.000	4.245	1.921	9.381
Drinking water before going to bed	0.965	0.406	5.651	0.017	2.624	1.184	5.813
Appetite	1.747	0.272	41.144	0.000	5.740	3.365	9.790
Stool frequency	2.260	0.596	14.387	0.000	9.586	2.981	30.826

### Comparison of response rates in different compliance groups of behavioral interventions

Complete response rate of enuresis (21.9%) and partial response rate (78.1%) in the complete compliancegroup were higher than those of the partial compliance group (complete response rate 11.3%, partial response rate 59.8%), the difference was statistically significant (P=0.000). The complete and partial response rate of the complete compliance group were higher than those of the non-compliance group (complete remission rate 0%, partial response rate 49.1%), the difference was statistically significant (P=0.000). The complete response rate and partial response rate of the partial compliance group were higher than those of the non-compliance group and the difference was statistically significant (P=0.003), as shown in Table-IV.

**Table-IV:** Comparison of response rates in different compliance groups of behavioral therapy.

*Groups*	*Complete response*	*Partial response*	*Non- response*
Complete compliance (n,%)	32(21.9%)P^a^P^b^	114(78.1%)P^a^P^b^	0(0.0%)P^a^P^b^
Partial compliance (n,%)	11(11.3%)P^c^	58(59.8%)P^c^	28(28.9%)P^c^
Non-compliance (n,%)	0(0.0%)	28(49.1%)	29(50.9%)

***Note:*** Adjusted a level = 0.0125, P <0.0125 was statistically significant.

a Compared with the partial compliance group P<0.0125

b Compared with the non-compliance group P<0.0125

c Compared with the non-compliance group P<0.0125

## DISCUSSION

Treatment methods of enuresis include behavioral interventions, enuresis alarm, and medications. Behavioral intervention is the backbone of enuresis treatment. There are some studies about factors prediction on the severity of enuresis but lack of analysis on specific life and behavioral factors. So we carried out this study to investigate the relationship between behavioral factors and the severity of enuresis. Multivariate analysis of data from patients revealed stool frequency to be an independent risk factor for predicting the severity of enuresis. As statedpreviously, urinary and bowel dysfunction are associated,^13^ because the bladder, urethraand rectum are closely connected to each other. If there is no stool for more than three days, rectal distension may compress the bladder wall directly, resulting in overactive bladder which plays an important role in the pathogenesis of enuresis. Ji Hyun et al.^14^ alsoreported that overactive bladder symptoms were improved by treating constipation. It can be seen that constipation is closely related to enuresis. Moreover, we found that appetite was an independent risk factor for the severity of enuresis. Children with poor appetite or fussy eaters are prone to be lack of iron, zinc, folic acid, vitamin B12, and other substances. These substances play key roles in the growth and development of children. Human infants with iron deficiency anemia showed lower in social-emotional, neurophysiologic, cognitive, and motor development than collation group infants.^15^ Furthermore, folic acid is an essential nutrient for central nervous system (CNS) development and zinc is beneficial to growth, intelligence development, and immunological function of children.^16^ From the above point of view, children with poor appetite are prone to development delays. According tovon GontardA et al.^17^, global CNS maturation delay may be a contributor to NE and development delays were common in children with enuresis. It was also found that vitamin B12 and folate needed for CNS maturation was lower among patients with PNE compared to the control group.^18^ Thus, the reason for children of poor appetite with severe symptoms may be nutrient insufficiency.

An underlying cause of NE is nocturnal polyuria. Nocturnal polyuria refers to increased urine production while asleep. The reasons for nocturnal polyuria may involve increased fluid intake prior to sleep and/or reduced production or response to antidiuretic hormone.^19^ The findings of this study indicated that drinking water before going to bed is an independent risk factor for the severity of enuresis. Drinking water before going to bed contributes to increasing the amount of urine at night and this may lead to increased frequency of enuresis. A number of wake-up treatments were compared by national clinical guidelines Center (NICE) of London in 2010^20^ and the results revealed that artificial wake group can result in 1.7 fewer wet nights per week compared with the placebo after 6-week treatment. Glazener^21^ suggested that star charts, with or without lifting or waking, were associated with significantly fewer wet nights and lower failure rates while on treatment. The results of this study are consistent with those of the above studies: non-awaking the child to toilet at night before treatment was associated with severe enuresis. In this study, we also compared remission rate in different compliance groups of behavioral interventions and found that the groups of better compliance had better therapeutic outcomes. Therefore, patients who appear treatment-resistant should be advised of the importance of full adherence and asked if they have had any difficulty with complying with recommendations.

## CONCLUSIONS

Stool frequency, drinking water before going to bed, awaking the child to toilet at night, and appetite are independent predictive factors affecting the severity of enuresis. Good compliance of behavioral interventions may have a crucial role for better therapeutic outcomes. Before and during the treatment, we should pay enough attention to behavior habits and behavioral interventions.

### Authors’ Contribution

**Yanli Ma:** Collected and analyzed the data, prepared the manuscript.

**Ying Shen and Xiaomei Liu:** Supervision and critical review of manuscript.
